# External Application of the Volatile Oil from *Blumea balsamifera* May Be Safe for Liver — A Study on Its Chemical Composition and Hepatotoxicity

**DOI:** 10.3390/molecules191118479

**Published:** 2014-11-13

**Authors:** Yu-Xin Pang, Zuo-Wang Fan, Dan Wang, Quan Yang, Kai Wang, Xiao-Lu Chen, Xuan Hu, Fu-Lai Yu, Zhen-Xia Chen

**Affiliations:** 1 Tropical Crops Genetic Resources Institute, Chinese Academy of Tropical Agricultural Sciences, Danzhou 571737, Hainan, China; E-Mails: fujianfanzuowang@126.com (Z.-W.F.); wang_dan1414@163.com (D.W.); jimojijie29@163.com (K.W.); hillowchan@hotmail.com (X.-L.C.); mchuxuan@163.com (X.H.); fulai.yu@163.com (F.-L.Y.); 18889584013@126.com (Z.-X.C.); 2 Key Laboratory of Crop Gene Resources and Germplasm Enhancement in Southern China, Danzhou 571737, Hainan, China; 3 School of Traditional Chinese Medicine, Guangdong Pharmaceutical University, Guangzhou 510006, Guangdong, China; E-Mail: yangquan7208@vip.163.com; 4 Hainan Provincial Engineering Research Center for *Blumea balsamifera*, Danzhou 571737, Hainan, China

**Keywords:** *Blumea balsamifera*, Ainaxiang, Nalong, Sambong, essential oil, chemical composition, hepatotoxicity

## Abstract

Ainaxiang (*Blumea balsamifera*), also known as Sambong, is an important ancient medicinal herb in Southeast Asia. It is rich in volatile oil, and still widely used nowadays for skin wound healing and treatment of sore throats. We analyzed the volatile oil from *Blumea balsamifera* (BB oil) by gas chromatography-mass spectrometry (GC-MS). Forty one components, including l-borneol, were identified. Next, the damaging effects of BB oil diluted with olive oil on liver at different concentrations (100%, 50%, 20%), were evaluated, using both normal and wounded skin. Plasma ALT, AST, ALP and TBili were assessed, along with liver histopathology. The results showed that serum levels of liver toxicity markers in the high concentration groups (100% w/v) increased compared with control groups, whereas no significant changes was observed in histopathology of liver samples. In the wound groups, treatment with BB oil resulted in a decrease in serum toxicity index, compared with normal animal groups. This study confirms the safety of short term BB oil consumption, though high BB oil doses may lead to mild liver injury and this response might be weakened in the case of cutaneous wounds. These results are expected to be helpful for guiding appropriate therapeutic use of BB oil.

## 1. Introduction

*Blumea balsamifera*, an ancient medicinal herb, grows preferentially in the wild throughout Southeast Asia. In China, it was called Ainaxiang and Nalong, and in some other Southeast Asian nations such as the Philippines, Sambong. It is the only source of l-borneol, an important Chinese Traditional Medicine. Other than that, it is well-known for its excellent effects on activation of blood circulation, wind-expelling and dampness dispersing, detumescence, traumatic injury, and sore healing [1]. In Chinese folk medicine, its leaves are directly applied on the forehead for headache relief and its infusion and decoction are commonly used for postnatal care, fever and stomach pain; it is also used as diuretic in hypertension and kidney stone treatment in the Philippines [2,3]. Recently, studies have also demonstrated that leaf extracts displayed antifungal [4], antibacterial [5,6], free radical-scavenging [7], superoxide radical scavenging [8], plasmin-inhibitory [9], anti-obesity [10] and anticancer [5,11,12] activities. In addition, an antidiarrheal activity was reported for *B. balsamifera* extracts by Indian authorities in 2013 [13].

The volatile oil from *B. balsamifera* (BB oil) has been used to make over-the-counter (OTC) medications, such as Jinhoujian spray, *etc.*, used in China for decades to treat throat sores and canker sores. Besides those with throat sores, some patients in China who had larynx and hypopharynx cancer have taken Jinhoujian spraying as a part of their treatment. What’s more, due to its unique scent, volatile oil from *B. balsamifera* has been used as a cosmetics additive. For example, gynecological lotions and shampoo liquid containing BB oils have been selling well in Southeast Asia for the last few years.

Phytochemical analysis of *B. balsamifera* leaves has revealed important amounts of volatile oil and a number of flavonoids, including velutin, dihydroquercetin-7,4′-dimethyl ether, blumeatin, ombuine, tamarixetin, rhamnetin, chrysoeriol, diosmetin, luteolin-7-methyl ether, luteolin, quercetin, 5,7,3′,5′-tetrahydroflavanone, dihydroquercetin-4′-methyl ether, 3,4,5-trihydroxy-3,7-dimethoxy-flavone, 3,4′,5-trihydroxy-3′,7-dimethoxyflavanone, 3′,4′,5-trihydroxy-7-methoxyflavanone and 3-O-7′′-biluteolin [14,15,16,17]. Moreover, five guaiane sesquiterpenes and one eudesmane sesquiterpene were identified in this plant [18]. The BB oil contains L-borneol, D-camphor, cineole, limonene, palmitic, myristic acid and sesquiterpene alcohol [19]. GC-MS analysis showed that this oil contains l-borneol (33.22%), caryophyllene (8.24%), ledol (7.12%) and phytol (4.63%) [20]. l-Borneol is a dominant component of the industrially produced *B. balsamifera* oil.

Interestingly, dihydroflavonol, extracted from *B. balsamifera*, was shown to display protective effects on liver and primary cultured hepatocytes against lipid peroxidation in rats [21,22]. In addition, Toshio *et al.* reported that the methanol extract of *B.* *balsamifera* was able to inhibit the growth of hepatocellular carcinoma [23]. Taken together, *B. balsamifera* contains different active constituents that may have antagonistic effects. For example, flavonoids are known to increase gastric motility (laxative effects) [24], whereas tannins can decrease gastric motility (constipating effects) [25]. Drinking of green tea is usually safe; however, the caplet / capsular form of pure green tea polyphenols can cause liver injury, especially in predisposing conditions, like fever [26]. Similarly, if a separated fraction of *B. balsamifera* is consumed rather than the whole herb, counteraction of harmful effects may be lost, causing biological effects that may not be consistent with the common usage of the whole *B. balsamifera* extract. The essential oil extracted from plants has been used in folklore medicine, and as cosmetics and food additives; however, it may cause hepatotoxicity or other adverse reactions [27,28].

Indeed, several questions remain unanswered [27,28]: Is the volatile oil from *B. balsamifera* safe for the liver after transdermal absorption? If it is, what is the maximum effective dose without significant toxicity and what is the role played by predisposing factors? If not, what is the adverse effect on liver and degree of hepatotoxicity? With these queries in mind, we initiated screening studies of different BB oil concentrations in rats to determine if any deleterious effects could be observed. Cutaneous wound is a frequent condition in which BB oil is used. To assess the influence of predisposing factors, we attempted to study the effect of BB oil under cutaneous wound conditions.

## 2. Results and Discussion

### 2.1. Results

#### 2.1.1. Phytochemical Analysis

A total of 49 components were found after phytochemical analysis of BB oil by GC-MS, of which 41 constituents were identified (Table 1).

**Table 1:** Chemical constituents of volatile oil from *Blumea balsamifera* (BB oil).

No.	RI ^a^	Constituent ^b^	Peak Area (%) ^c^
1	814	(*E*)-2-Hexenal	0.04
2	860	1-Hexanol	0.21
3	868	(3 *E*)-3-Hexen-l-ol	0.11
4	868	(*E*)-2-Hexen-l-ol	0.08
5	874	*n*-Butyl acrylate	0.26
6	943	β-Pinene	5.2
7	943	Camphene	1.5
8	948	α-Pinene	2.04
9	952	3-Octanone	0.21
10	958	β-Myrcene	0.1
11	969	1-Octen-3-ol	8.31
12	976	(*E*)-β-Ocimene	0.19
13	976	(*Z*)-β-Ocimene	0.76
14	979	3-Octanol	1.36
15	1018	(+)-Limonene	0.39
16	1042	o-Cymene	0.24
17	1059	1,8-Cineole	0.06
18	1072	Hotrienol	0.28
19	1082	Linalool	1.76
20	1119	Chrysanthenone	0.22
21	1121	D-Camphor	9.54
22	1137	Terpinenol-4	0.09
23	1138	l-Borneol	43.55
24	1143	α-Terpinenol	0.24
25	1207	(−)-Perillaaldehyde	0.13
26	1230	Cuminaldehyde	0.1
27	1277	Bornyl acetate	0.41
28	1351	1,3,4,5,6,7-Hexahydro-2,5,5-trimethyl-2*H*-2,4a-ethanonaphthalene	2.93
29	1386	2-*tert*-Butyl-1,4-dimethoxybenzene	3.07
30	1387	Longifolene-(V4)	2.14
31	1396	Dehydroaromadendrene	0.11
32	1419	(−)-α-Gurjunene	0.22
33	1435	(−)-γ-Cadinene	0.2
34	1462	Alloaromadendrene oxide-(1)	0.34
35	1494	(*E*)-β-Caryophyllene	6.51
36	1507	Caryophyllene oxide	0.89
37	1530	Ledol	0.12
38	1579	β-Caryophyllene	1.54
39	1614	Guaiol	0.41
40	1626	γ-Eudesmol	0.33
41	1628	Xanthoxyline	1.4
		Sum	97.59

^a^ IR: Retention indices on DB-5 ms column in reference to n-alkanes (C_6_-C_32_); ^b^ The compounds were identified by comparison of their GC retention indices and comparison of their MS spectra with those of standard compounds available in the laboratory (l-Borneol, d-Camphor, β-pinene, β-caryophyllene), and by matching mass spectral data with those from the National Institute of Standards and Technology (NIST 08, NITS 08s) database; ^c^ Area%: Peak area obtained by GC-FID.

#### 2.1.2. Effect of Different Concentrations of BB Oil on Plasma ALT Levels in Normal and Wounded Rats

**Figure 1:**
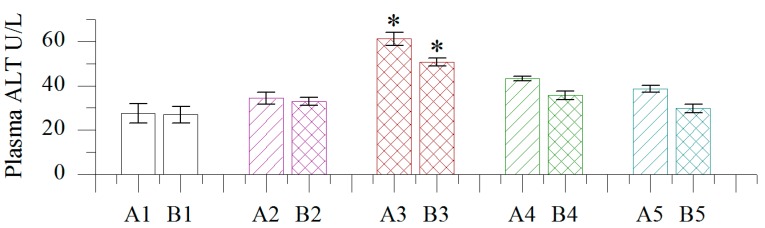
Effect of different concentrations of volatile oil from *Blumea balsamifera* (BB oil) on plasma ALT levels in rats. 1–5: CK, Olive oil, 100% BB oil, 50% BB oil, 20% BB oil; A, B: normal skin, wound skin; *: Columns labeled by * are significantly different from others by Duncan’s test at *p* < 0.05.

As shown in Figure 1, only TDD of the highest BB oil concentration (100 w/v) resulted in significant increase of plasma ALT levels compared to control and vehicle groups, both for the normal and wound bearing animals. All the other treatments did not show any significant shift in ALT levels. Generally, ALT levels were lower in the wound groups in comparison with normal groups.

#### 2.1.3. Effect of Different Concentrations of BB Oil on Plasma AST Levels in Normal and Wound Bearing Rats

**Figure 2:**
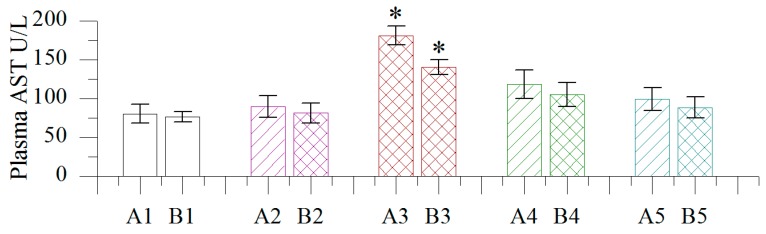
Effect of different concentrations of volatile oil from *Blumea balsamifera* (BB oil) on plasma AST levels in rats. 1–5: CK, Olive oil, 100% BB oil, 50% BB oil, 20% BB oil; A, B: normal skin, wound skin; *: Columns labeled by * are significantly different from others by Duncan’s test at *p* < 0.05.

As shown in Figure 2, plasma AST levels increased with BB oil treatment at all concentrations. As observed with ALT levels, the highest BB oil concentration (100 w/v) resulted in significantly increased plasma AST levels compared to control and vehicle groups. However, AST levels were still within normal range in all groups. 

#### 2.1.4. Effect of Different Concentrations of BB Oil on Plasma ALP Levels in Normal and Wound Bearing Rats

**Figure 3:**
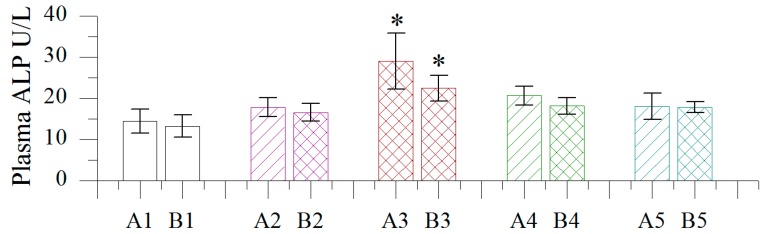
Effect of different concentrations of volatile oil from *Blumea balsamifera* (BB oil) on plasma ALP levels in rats. 1–5: CK, Olive oil, 100% BB oil, 50% BB oil, 20% BB oil; A, B: normal skin, wound skin; *: Columns labeled by * are significantly different from others by Duncan’s test at *p* < 0.05.

As shown in Figure 3, TDD of BB oil at different concentrations resulted in no significant shift in plasma ALP levels compared to control and vehicle groups, except for the highest BB oil concentration (100% w/v).

#### 2.1.5. Effect of Different Concentrations of BB Oil on Plasma Total Bilirubin Levels in Normal and Wound Bearing Rats

**Figure 4:**
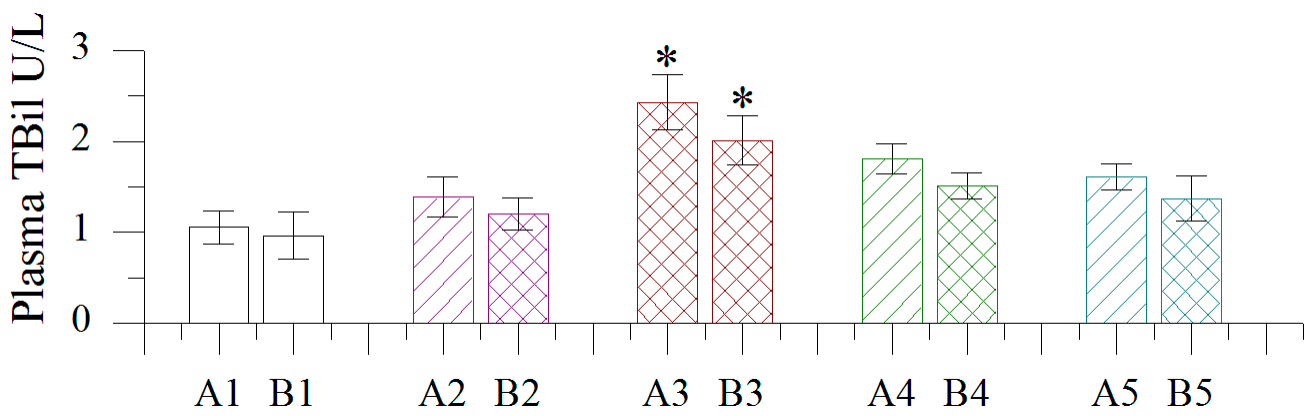
Effect of different concentrations of volatile oil from *Blumea balsamifera* (BB oil) on plasma TBili levels in rats. 1–5: CK, Olive oil, 100% BB oil, 50% BB oil, 20% BB oil; A, B: normal skin, wound skin; *: Columns labeled by * are significantly different from others by Duncan’s test at *p* < 0.05.

As shown in Figure 4, TBili levels followed the same trend observed with ALP. Of all BB oil concentrations administered by TDD, only the highest amount (100% w/v) showed a significant shift in plasma TBili levels compared to control and vehicle groups.

#### 2.1.6. Histopathology of Liver Samples after Treatment with Different Concentrations of BB Oil in Normal Rats

**Figure 5:**
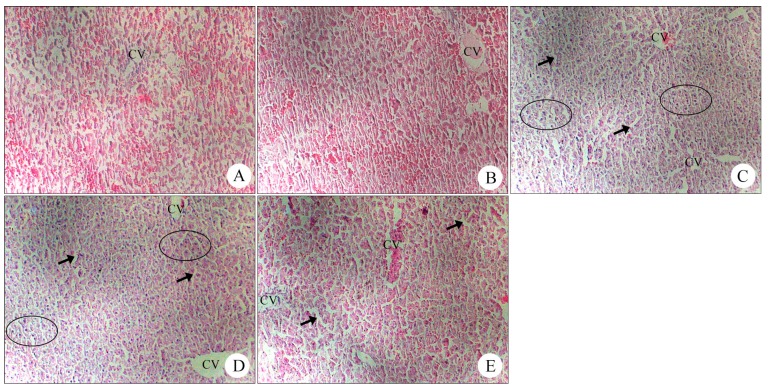
Histopathology of liver samples after treatment with different concentrations of volatile oil from *Blumea balsamifera* (BB oil) in normal rats. (**A**) Group A1: Control (the blank group) showed normal liver structure. (**B**) Group A2: Olive oil (vehicle) showed diffused vacuolar changes. (**C**) Group A3: BB oil (100% w/v) 1000 mg/kg showed diffused vacuolar changes; hepatocytes appeared pale with small discrete cytoplasmic vacuoles (arrow). Hepatocytes showed clear cytoplasm and pyknotic nuclei (circles), suggesting early mild hepatocellular necrosis. (**D**) Group A4: BB oil (50% w/v) 1000 mg/kg showed diffused vacuolar changes; hepatocytes appeared pale with small discrete cytoplasmic vacuoles or foamy cytoplasm (arrow); hepatocytes showed cytoplasm and pyknotic nuclei (circles). However, cytoplasmic vacuoles and pyknotic nuclei were significant less than in Group A3. (**E**) Group A5: BB oil (20% w/v) 1000 mg/kg showed diffused vacuolar changes; hepatocytes appeared pale with small discrete cytoplasmic vacuoles (arrow).

Histopathological analysis did not reveal any remarkable lesions after treatment with BB oil at 100%, 50% and 20% w/v in normal rats (Figure 5). However, liver sections from rats treated with 100% w/v showed minor alterations, including hepatocellular vacuolar changes and increased Kupffer cell numbers. Of note, the presence of these lesions does not reflect severe liver injury.

#### 2.1.7. Histopathology of Liver Samples after Treatment with Different Concentrations of BB Oil in Wound Bearing Rats

**Figure 6:**
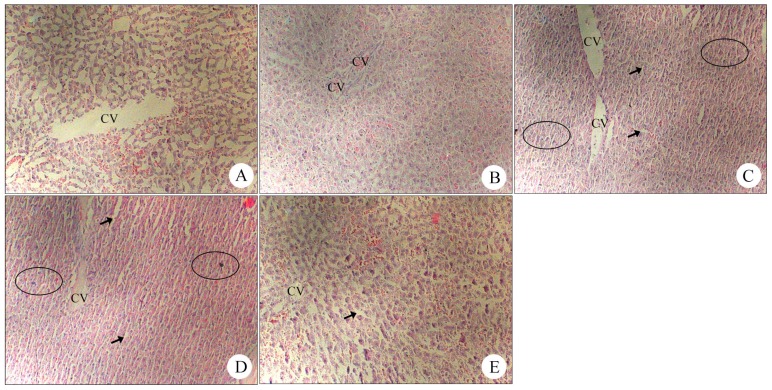
Histopathology of liver samples after treatment with different concentrations of volatile oil from *Blumea balsamifera* (BB oil) wound bearing rats. (**A**) Group B1: Control (the blank group) showed normal liver structure. (**B**) Group B2: Olive oil (vehicle) showed diffused vacuolar changes. (**C**) Group B3: BB oil (100% w/v) 1000 mg/kg showed diffused vacuolar changes; hepatocytes appeared pale with small discrete cytoplasmic vacuoles (arrow), displaying clear cytoplasm and pyknotic nuclei (circles). These results suggested an early mild hepatocellular necrosis; however, the lesions were reduced compared with A3 (normal group). (**D**) Group B4: BB oil (50% w/v) 1000 mg/kg showed diffused vacuolar changes and appeared pale with small discrete cytoplasmic vacuoles (arrow). Moreover, some hepatocyte pyknotic nuclei were observed (circles). (**E**) Group B5: BB oil (20% w/v) 1000 mg/kg showed diffused vacuolar changes; hepatocytes appeared mild cytoplasmic vacuoles (arrow) and slight amounts of pyknotic nuclei (circles).

Histopathological analysis did not reveal any remarkable lesions after treatment with BB oil at 100% w/v, 50% w/v, 20% w/v in the wound groups (Figure 6).

### 2.2. Discussion

*B. balsamifera* is the only plant source for l-borneol extraction recorded in the Chinese Pharmacopoeia. It is also an important medicinal herb among the Miao and Li nationality. BB oil can be obtained not only by steam distillation but also as a by-product after extraction of l-borneol from the plant. This volatile oil, which is mainly composed of l-borneol, d-camphor, 1-octen-3-ol, and (*E*)-β-caryophyllene, is currently used to make OTC medications and, with special odor, as a cosmetic additive. This study aimed to evaluate the side effects of BB oil so as to assess the safety of these products.

In this short-term study, TDD of BB oil at three different concentrations 100%, 50% and 20% did not cause any significant effect on the liver in normal animal groups, as observed with the levels of ALT, AST, ALP and TBili. These results were confirmed by histopathology analysis of liver samples, which showed no significant lesions, except for some minor vacuolar changes and increased Kupffer cell numbers, especially in the high concentration group (100% w/v). These lesions are of uncertain significance but don’t provide unequivocal evidence of major hepatotoxicity. Hepatocellular vacuolation is indicative of altered hepatocellular metabolism [29]. Mild changes may also indicate nutritional influences [30]. It must be borne in mind that 100% BB oil is a very high dose for rats and does not correspond to the normal use of BB oil as cosmetic additives in humans. However, this corresponds to the direct consumption of the essential oil in special circumstances. In order to make BB oil safe for human use, future studies are needed to clearly define the composition of BB oil containing products and the role they may play in index changes with reference to BB oil consumption.

The skin was damaged in the wound groups, and this resulted in decreased absorptivity. In wound animal groups, we demonstrated that TDD of BB oil at three different concentrations (100%, 50% and 20%) did not cause any significant effect on liver as observed with the levels of ALT, AST, ALP and TBili. All concentrations of BB oil showed a decrease in serum index compared to the normal animal groups, suggesting that wound affected skin absorptivity. These results were confirmed with histopathology analysis of liver samples, where no significant lesions or acute hepatotoxicity were observed.

In our recent study, 100% w/v BB oil applied to intact and damaged skin for 24 h showed no significant allergy or acute toxicity [31]. This study was carried out to further evaluate the short term adverse reactions of human consumption of BB oil. The levels of serum index and liver histopathology confirmed the safety of high BB oil concentrations. In addition, our data confirmed that no significant positive correlation exists between BB oil absorption and concentration. However, further investigations are required to clarify this issue.

Since essential oils have good solubility in vegetable oil, the vehicle used herein was olive oil instead of DMSO or other organic solvents [32,33,34]. It tends to completely dissolve and diffuse the BB oil. Olive oil, which is often employed in cosmetic dermatology, has been reported to have a stimulating effect on skin wound healing [35,36,37]. In wound animal groups, wounds treated with 50% w/v concentration of BB oil showed significantly decreased time of wound healing compared with other groups. The wound-healing property of BB oil may be attributed to L-borneol that has been shown to accelerate skin absorption [38,39]. To sum up, short term external application of the volatile oil from *B. balsamifera*, diluted into certain concentrations by olive oil, may be safe for liver. 

## 3. Experimental Section

### 3.1. Plant Material

*B. balsamifera* plants were collected in Luodian (Guizhou, China), one of the most important producing areas of this medical plant, and authenticated by Prof. Zhu-Nian Wang. Voucher specimens (TCGRI 10011) have been deposited at the Traditional Medicinal Plant Germplasm Nursery of South China, Tropical Crops Genetic Resources Institute, Chinese Academy of Tropical Agricultural Sciences, Hainan Province, China.

### 3.2. Volatile Oil Obtaining

The volatile oil was isolated by hydrodistillation at the Guizhou Ai-Yuan Ecological Medicine Development Industry (Guizhou, China). The leaves of *B. balsamifera* were air-dried before steam distillation. The oil yield of fresh *B. balsamifera* leaves was about 0.01%. The volatile oil was stored in sealed amber ampules at 4 °C until being tested or analyzed.

### 3.3. GC-FID Analysis

The volatile oil was analyzed on a GC-2010 instrument equipped with GCMS solution Ver. 2.5 software (Shimadzu, Tokyo, Japan), a flame ionization detector (FID) and a DB-5 ms capillary column (30.0 m × 0.25 mm; film thickness, 0.25 µm). The injector temperature was maintained at 250 °C. The oven temperature was programmed from 50 °C for 2 min, raised to 180 °C at a rate of 2 °C /min, and isotherm at 180 °C for 4 min. Helium was the carrier gas, at a flow rate of 1 mL/min. A sample of 0.1 mL of volatile oil was injected (in split mode 20:1). Volatile oil components were calculated as a relative percent of the total oil by peak area.

### 3.4. GC-MS Analysis

The volatile oil was analyzed on a GCMS-QP2010 Plus Mass Spectrometer (Shimadzu) equipped with a DB-5 ms capillary column (30.0 m × 0.25 mm; film thickness, 0.25 μm) and mass spectrometry MS detector (MS). GC conditions were the same as described above. Acquisition parameters were full scan at scan range 40–350 amu*.*

### 3.5. Identification of Constituents of Essential Oils

Individual identification of the constituents was accomplished by comparison of their GC retention indices determined with reference to a homologous series of normal C_6_–C_32_ alkanes and comparison of their GC retention times with those of standard compounds available in the laboratory (l-borneol, d-camphor, β-pinene, β-caryophyllene), and of their MS spectra by matching the mass spectral data with those from the National Institute of Standards and Technology Mass Spectra Library data (NIST 08, NITS 08s, National Institute of Standards and Technology, Gaithersburg, MD, USA). The oil compositions are presented in Table 1.

### 3.6. Experimental Animals

Healthy Sprague-Dawley rats (220–250 g) were housed in polypropylene cages and maintained in standard laboratory conditions of temperature (24 ± 2 °C) and light-dark cycle (12 h/12 h). They were allowed standard pellet diet and water *ad libitum*. Before any treatment, rats were housed for 7 days to allow adaptation. At the end of the experiment the animals were sacrificed under anesthesia.

### 3.7. Animal Model

The dorsal fur of anesthetized animals was shaved with surgical scissors on an area of 4 cm × 7 cm. Then, animals were randomly divided into normal and wound groups. In animals of the wound groups, a “#” square wound was created in each animal (area 200 mm^2^ and 2 mm depth) with a surgical blade. All animals received BB oil treatment (Table 2 and Table 3).

**Table 2:** Normal animal groups (*n* = 5/group): treatments, doses and route of administration of different concentrations of volatile oil from *Blumea balsamifera* (BB oil) concentrations.

Group No.	Treatments	Doses (mg/kg)
A1	Control	0
A2	Olive oil (vehicle)	1000
A3	BB oil (100% w/v)	1000
A4	BB oil (50% w/v)	1000
A5	BB oil (20% w/v)	1000

**Table 3:** Wound animal groups (*n* = 5/group): treatments, doses and route of administration of different concentrations of volatile oil from *Blumea balsamifera* (BB oil) concentrations.

Group No.	Treatments	Doses (mg/kg)
B1	Control	0
B2	Olive oil (vehicle)	1000
B3	BB oil (100% w/v)	1000
B4	BB oil (50% w/v)	1000
B5	BB oil (20% w/v)	1000

### 3.8. Sample Collection

Blood samples were collected from rats under anesthesia by vein puncture in heparinized tubes, and centrifuged at 4000 r/min for 15 min at 15 °C for plasma collection. The plasma samples were stored at −80 °C in aliquots until analysis. Liver tissue samples (left median lobe) were extracted and processed by standard histological techniques.

### 3.9. Clinical Chemistry

Plasma levels of alanine aminotransferase (ALT), aspartate aminotransferase (AST), alkaline phosphatase (ALP) and total bilirubin (TBili) were measured spectrophotometrically using commercial diagnostics kits (Nanjing Jiancheng Bioengineering Institute, Nanjing, China).

### 3.10. Histopathologic Evaluation

Livers were processed routinely and embedded in paraffin blocks. Liver sections were prepared (5 µm) and stained with hematoxylin and eosin (H&E). The slides were assessed for liver injury using a light microscope (×200).

### 3.11. Statistical Analysis

The results were expressed as mean ± SD (standard deviation). Data were analyzed by one-way ANOVA followed by Duncan’s multiple range tests using Statistical Package for Social Sciences 16.0 (SPSS, Chicago, IL, USA). A *p*-value of less than 0.05 was considered statistically significant.

## 4. Conclusions

This study showed that TDD of *B. balsamifera* oil did not cause short-term liver injury in either normal or skin wound animals. It increased ALT, AST, ALP and TBili concentrations. However, liver sample histopathology revealed no significant lesions, and only minor changes were observed. These findings confirm the safety of cosmetic products containing BB oil as an additive. However, these findings suggest that high concentrations (100% w/v) of BB oil can lead to mild liver injury and under cutaneous wound conditions, this response might be weakened. Further studies are needed to isolate the active ingredients of BB oil that are responsible for its biological activities.

Though whether administration of BB oil would promote wound healing has yet to be studied, and the mechanism underlying this effect needs further investigation, the present results indicate that proper use of certain concentrations of BB oil may be safe for the liver. As *B. balsamifera* has been wildly used for centuries without sufficient toxicology studies [40], and our former research has shown that the external application of *B. balsamifera* oil (2000 mg/kg, 24 h) on intact and damaged skin of rats exhibited no acute toxicity [31], this study may add more laboratory data to this field.
